# Increased Gut Permeability and Bacterial Translocation after Chronic Chlorpyrifos Exposure in Rats

**DOI:** 10.1371/journal.pone.0102217

**Published:** 2014-07-14

**Authors:** Claire Joly Condette, Hafida Khorsi-Cauet, Patrice Morlière, Luciane Zabijak, Julie Reygner, Véronique Bach, Jérôme Gay-Quéheillard

**Affiliations:** 1 Peritox Laboratory, EA4285 UMI01 Ineris, Faculty of Medicine, Jules Verne University of Picardy, Amiens, France; 2 INSERMU1088, Faculty of Medicine, Jules Verne University of Picardy, Amiens, France; 3 Biochemistry Laboratory, Human Biology Centre, Amiens University Hospital, Amiens, France; 4 EA4666, Jules Verne University of Picardy, Amiens, France; National Institute of Agronomic Research, France

## Abstract

The epithelium's barrier function is crucial for maintaining homeostasis and preventing the passage of food antigens and luminal bacteria. This function is essentially subserved by tight junctions (TJs), multiprotein complexes located in the most apical part of the lateral membrane. Some gastrointestinal disease states are associated with elevated intestinal permeability to macromolecules. In a study on rats, we determined the influence of chronic, daily ingestion of chlorpyrifos (CPF, a pesticide that crosses the placental barrier) during pre- and postnatal periods on intestinal permeability and TJ characteristics in the pups. Fluorescein isothiocyanate (FITC)-dextran was used as a marker of paracellular transport and mucosal barrier dysfunction. Pups were gavaged with FITC-dextran solution and blood samples were collected every 30 min for 400 min and analyzed spectrofluorimetrically. At sacrifice, different intestinal segments were resected and prepared for analysis of the transcripts (qPCR) and localization (using immunofluorescence) of ZO-1, occludin and claudins (scaffolding proteins that have a role in the constitution of TJs). In rats that had been exposed to CPF *in utero* and after birth, we observed a progressive increase in FITC-dextran passage across the epithelial barrier from 210 to 325 min at day 21 after birth (weaning) but not at day 60 (adulthood). At both ages, there were significant changes in intestinal TJ gene expression, with downregulation of ZO-1 and occludin and upregulation of claudins 1 and 4. In some intestinal segments, there were changes in the cellular localization of ZO-1 and claudin 4 immunostaining. Lastly, bacterial translocation to the spleen was also observed. The presence of CPF residues in food may disturb epithelial homeostasis in rats. Changes in TJ protein expression and localization may be involved in gut barrier dysfunction in this model. Uncontrolled passage of macromolecules and bacteria across the intestinal epithelium may be a risk factor for digestive inflammatory diseases.

## Introduction

The intestinal epithelium constitutes the host's first line of defense against exogenous agents such as food antigens, live bacteria, bacterial products and other exogenous agents present in the intestinal lumen.

At birth, the epithelial barrier becomes more selective: nutrients are absorbed but potentially harmful intraluminal bacteria, microbial products and exogenous chemical substances are excluded [1].

Excessive intestinal permeability to macromolecules may favor the uncontrolled passage of antigens and the onset of intestinal inflammation or gastrointestinal disease [2].

The integrity of the protective epithelial cell layer is primarily maintained by intercellular junctional complexes (such as tight junctions (TJs) [3], adherens junctions and desmosomes), whereas gap junctions provide channels for intercellular communication. Tight junctions are the most apical membrane proteins complex and are primarily involved in the regulation of paracellular permeability and membrane polarity. Extensive research over the past two decades has identified the TJ's molecular constituents and the latter's interactions. Tight junctions are composed of several transmembrane proteins (occludin and the claudins) and intracellular proteins. Occludin and claudins family are linked to cytoskeletal components by the zona occludens (ZO) family of proteins (ZO-1, ZO-2 and ZO-3); which connects the TJs to other cell-cell and cell-substratum adhesion sites. Recent data suggest that the TJ is a dynamic, multimolecular, multifunctional complex. Occludins are also thought to have a regulatory role, where claudins are mainly structural [3].

In the intestine, the interplay between gut bacterial colonization, the epithelial barrier and the immune system has a crucial role in the development of intestinal function and maturation of gut-associated lymphoid tissue (GALT) during the first months of life in human neonates and the first weeks of life in rodents. It has also been shown that bacterial translocation is required to achieve GALT maturation in the neonate [4]. This interaction can be modulated by other factors, such as food components and food contaminants. Indeed, food can contain pesticide residues that may modulate immune maturation, intestinal microbiota and/or epithelial barrier function. By way of an example, a recent study of a piglet model of digestive immaturity at birth found that feeding with a high-protein formula milk was associated with greater ileal permeability [5].

Altered intestinal permeability and structure during the neonatal period may have immediate and/or long-term health impacts. It is increasingly accepted that intestinal barrier dysfunction in neonates (i) contributes to adverse clinical outcomes, and (ii) induces susceptibility to infection, inflammation, hypersensitivity and stress in both childhood and adulthood [6], [7].

Chlorpyrifos (O,O-diethyl-O-[3,5,6-trichloro-2-pyridinyl]phosphorothioate: CPF) is an organophosphate insecticide used worldwide to treat fruit and vegetable crops. Chlorpyrifos residues are often detected in food and drinking water [8]. In the European Union, the maximum residue limit (MRL) for CPF in food corresponds to an average intake of about 1 mg/day in humans (European Union 2005).

Although the digestive tract is the first organ to come into contact with food contaminants, little is known about CPF's impact on the epithelial barrier. Two studies have shown that CPF increases intestinal permeability: an *ex vivo* model using the single-pass intestinal perfusion method (with exposure to 0.1, 2 and 10 µM CPF for 2 h) [9] and an *in vitro* model based on an enterocyte cell line (with exposure to 30, 50 and 250 µM CPF) [10]. In contrast, CPF's impact on intestinal permeability after chronic *in utero* and postnatal exposure has not previously been studied. This topic is of particular interest because CPF is known to cross the placental barrier.

The present study was designed to evaluate the impact of *in utero* and postnatal oral low-dose CPF administration on gut maturation. We focused our investigation on gut epithelial permeability, TJ protein expression and bacterial translocation.

## Materials and Methods

### 1. Chemicals

Chlorpyrifos (purity: 99.8%) was purchased from LGC Standards (Molsheim, France). It was dissolved in commercially available rapeseed oil as vehicle and administered by gavage (at a dose of 1 mg/kg bodyweight/day) to the animals in the CPF1 group. This dose corresponds to (i) the maximum daily intake and (ii) the oral “no observed effect level” for maternal toxicity, as indicated by the inhibition of cholinesterase activity [11]. Animals in a control (CPF0) group were gavaged with a volume of 1 mL/kg bodyweight of vehicle.

The high-molecular-weight fluorescent marker fluorescein isothiocyanate-dextran 4 kDa (FITC-Dextran) powder was purchased from Sigma Aldrich (Saint Louis, MO, USA) and dissolved in pure water to a concentration of 80 mg/mL.

### 2. Animals

All animal experiments were approved by the Animal Care and Use Committee at Jules Verne University of Picardy (Amiens, France; file reference: #2011/A/1). Thirteen female and eleven male Hannover Wistar rats (age on delivery: 8 weeks; body weight range: 215–300 g) were obtained from Janvier (Le Genest St Isle, France). Animals were housed in cages in a temperature-controlled room (23°C), with a constant relative humidity of 26% and artificial illumination (a 12-h dark/light cycle). They were maintained on a standard pelleted diet, with tap water *ad libitum*. After a one-week acclimation period, females were mated with males (two females per male). On the first day, each male was placed with the females overnight (O/N). The presence of spermatozoa in the vagina was checked with a smear morning and evening on the following days) [12]. After fertilization, 13 pregnant females (mean age: 18±1 weeks; body weight range: 288±12 g) were individually housed in clean plastic cages and randomly assigned (1∶1) to a treatment group (n = 7) or a control group (n = 6).

### 3. Experimental conditions

The day of parturition was considered to be postnatal day 0 (PND0). From days 0 to 21 of gestational period, dams received daily doses of 1 mg CPF/kg bodyweight (in the CPF1 group) or vehicle (in the CPF0 group). Pups were thus exposed *in utero* and then postnatally through their maternal milk (from PND0 to D21).

At PND1, each pup's legs were tattooed with a special veterinary ink (Centravet, Nancy, France) for identification and monitoring. The pups in each litter were counted, sexed, and weighed at PND0 (CPF0, n = 26; CPF1, n = 30). The animal body weight was recorded at PND1, 2, 5, 7, 10, 13, 16, 19, and 21.

Two different developmental time points were studied: D21 (weaning) and D60 (adulthood). At D21, intestinal permeability was studied in some pups (CPF0: n = 12; CPF1: n = 17). The first blood sample was collected immediately prior to administration of FITC-dextran, as a negative control for plasma background fluorescence. The pups were then gavaged with FITC-dextran solution (60 mg/100 g body weight). Blood samples were collected 30 min after FITC-dextran administration and then every 30 min until end of experiment at 400 min. Blood was drawn by tail vein punctures on anesthetized rats (Animals were anesthetized during all experiment (40% Ketamine 1000, 25% Xylazine 2%, 0,1 mL/100 g body weight). At the end of the experiment, rats were euthanized by intraperitoneal administration of sodium pentobarbital (100 mg/kg; 200 mg/mL solution from Ceva Santé Animale (Libourne, France)) and different intestinal segments (ileum and colon) and organs (spleen) were removed under sterile conditions.

The other pups were weaned, separated from their mothers, and divided into groups (with three pups per cage) until the age of 60 days (CPF0: n = 14; CPF1: n = 13). From D21 to D60, the animals were individually gavaged with either vehicle alone (for CPF0) or 1 mg/kg/day CPF (as previously described for the dams). At D60, intestinal permeability was investigated in the same way as at D21 and the animals were then all euthanized. Again, different intestinal segments (ileum and colon) and sterile organs (spleen) were removed under sterile conditions. Given that similar patterns were obtained for all sterile organs, only results for the spleen are presented below.

### 4. Fluorometric assay of FITC dextran

Intestinal permeability was evaluated in terms of the amount of FITC-dextran that had crossed the intestinal epithelial barrier into the blood after oral ingestion. Serum FITC-dextran levels were estimated every 30 min during 400 min by fluorometric determination. After a five-fold dilution in PBS (phosphate buffered saline), the sample emission spectrum from 600 to 480 nm was recorded upon excitation at 475 nm. Emission and excitation slits were set so that the emission or excitation peak width at half-height was 3 nm. Long-wave pass filters (Métallisations et Traitements Optiques, Massy, France) were placed in the excitation path (J400) and the emission path (J423). The second derivatives of these spectra were then calculated. Second-derivative spectroscopy is a useful technique for eliminating background noise and light scattering. The FITC dextran concentration corresponded to the height of the peak at 514 nm. A calibration curve for FITC dextran in serum was found to be linear up to 0.01 µg/µL.

### 5. Epithelial barrier integrity assays

#### 5.1 Real-time PCR

Total RNA was extracted from intestinal tissues by using the RNeasy Mini Kit (Qiagen, Austin, Texas, USA), according to the manufacturer's instructions. The mRNA content was determined spectrophotometrically using a NanoDrop system (Thermoscientific, Wilmington, USA). Complementary DNA (cDNA) was generated by reverse transcription of 1 µg mRNA withahigh-capacity cDNA reverse transcription kit containing RNase inhibitor (Life Technologies SAS, Saint Aubin, France) according to the manufacturer's instructions.

The real time quantitative polymerase chain reaction was run on 96-well PCR microplates (Life Technologies SAS, Saint Aubin, France) containing 1 µL cDNA, 1 µL TaqMan gene expression assay primers, 8 µL UltraPure DEPC-Treated Water (Invitrogen, Life Technologies SAS, Saint Aubin, France), 10 µL TaqMan gene master mix in a total of 20 µL per sample per well.

Primers were provided by Life Technologies: Rn02116071_s1 for ZO-1 (Tjp1; amplicon length 156 bp), Rn00580064_m1 for occluding (amplicon length 86 bp), Rn00581740_m1 for claudin 1 (Cldn1; amplicon length 113 bp), Rn01196224_s1 for claudin4 (Cldn4; amplicon length 86 bp), Rn99999916_s1 for the endogenous control glyceraldehyde 3-phosphate dehydrogenase (GAPDH) (Gapdh; amplicon length 87 bp), Rn00667869_m1 for the endogenous control beta-actin (beta-act; amplicon length 91 bp). For Tjp1, Cldn4 and Gapdh, the primers and the probe all map within a single exon. For Cldn1, occludin and beta-actin, the probe spans exons.

The PCRs were run on a thermal cycler (StepOnePlus, Lifetechnologies SA, France), according to the manufacturer's instructions. The cycle numbers at the respective linear amplification thresholds of the control GAPDH gene and the target gene were recorded.

Relative expression of the target gene (normalized against the control gene) was calculated using the comparative Ct method: 2^−(ΔCt)^ [13].

#### 5.2 Immunohistochemical analysis

After sacrifice, segments of the jejunum, ileum and colon were snap-frozen after embedding in water-soluble glycol resin (O.C.T. reagent, Q Path, VWR International, Templemars, France) and stored at −80°C. Ten-micrometer sections were cut with a microtome cooled to −20°C. The sections were then placed on slides at room temperature (to promote adhesion) and then refrozen at −80°C until further use. Slides were thawed at room temperature for 15 min, incubated in 100% cold acetone (4°C) for 5 min and then washed three times in cold PBS (4°C). To block endogenous peroxidase activity, the slides were incubated in 3% hydrogen peroxide for 10 min and then rinsed twice in PBS. Sections were incubated for 60 min at room temperature in a blocking solution containing 1% bovine serum albumin and 5% normal goat serum in PBS. Primary antibodies for ZO-1 (rabbit anti ZO-1, 61-7300, Invitrogen, Camarillo, CA, USA) and for Cldn4 (rabbit polyclonal IgG, C-18 sc-17664-R, Santa Cruz Biotechnology, Heidelberg, Germany) were used at a dilution of 1∶50 in blocking buffer and incubated O/N at 4°C. For negative controls, the right half of each slide was incubated in blocking buffer only. After O/N incubation, slides were washed three times in PBS to remove primary antibodies. An FITC-conjugated goat anti-rabbit secondary antibody (F0382, Sigma) diluted 1∶500 in PBS was then applied to the slides for 60 min at room temperature in the dark. The slides were then washed three times in PBS, coverslipped with DAPI-containing UltraCruz mounting medium for fluorescence (Santa Cruz Biotechnology) and stored at 4°C in a black box. Analyses were performed using a fluorescence microscope (Zeiss, Marly le Roi, France). Five animals from each group were studied at each time point (i.e. five CPF0 and five CPF1 animals at D21, and five CPF0 and five CPF1 animals at D60).

### 6. Bacterial translocation

After euthanasia, the rat skin was washed with 70% ethanol before opening the abdomen. Sterile organs (spleen) were removed under aseptic condition and cultured for translocated bacteria. Each portion of thawed tissue was placed in a sterile, pre-weighed glass.

On the day of inoculation, organs were weighed, placed in a sterile blending bag and homogenized in Ringer's saline solution (Bio-Rad, France). Plates were inoculated with the resulting homogenate (100 µL of diluted homogenate per plate) and cultured (at 37°C) either anaerobically for 72 h or aerobically for 48 h. The conventional microbial media used in this experiment have been previously described by Joly et al. [14].

After incubation, bacteria were identified using standard microbiological techniques [15]. Colony counting and bacterial identification were performed in a blind manner. The viable bacterial counts were expressed as the log10 mean ± standard error of the mean (SEM) number of colony forming units per gram of tissue.

### 7. Statistical analysis

Values are expressed as the mean±SEM. Intergroup comparisons were performed using a Kruskal–Wallis test. If the Kruskal–Wallis test result was statistically significant, a Mann–Whitney test was then performed. The threshold for statistical significance was set to p<0.05 for all tests. Only the p value for the Mann-Whitney test is reported below. Translocation data were analyzed using a χ2 test for comparison of scores (positive *vs.* negative samples) between groups.

All analyses were performed with StatView software (version 5.0, Abacus Concepts Inc., Berkeley, CA, USA).

## Results

### Growth

**Figure 1:**
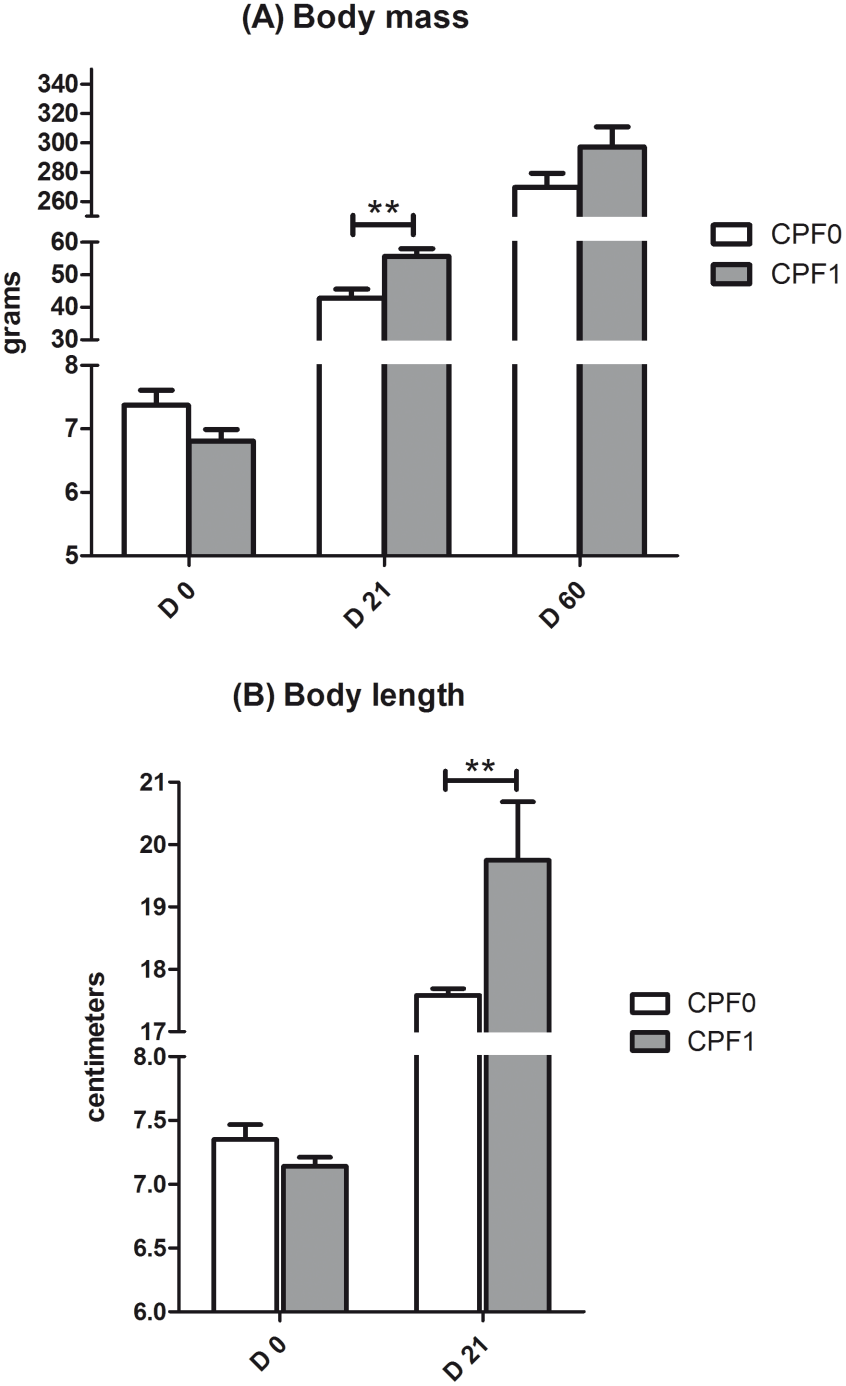
Body mass (g) (A) and body length (cm) (B) of control rats (CPF0, open bars) or CPF-exposed pups (CPF1, 1 mg/kg/day, grey bars) at birth (D0), at weaning (D21) and as young adults (D60). Values are quoted as the mean ± SEM. D0: n = 41, CPF1 n = 37; D21: CPF0 n = 28, CPF1 n = 24; D60: CPF0 n = 21, CPF1 n = 13. ** p<0.01.

To establish whether or not CPF induced a growth delay, we weighed and measured the pups at birth, at weaning and at D60 (Figure 1).

At weaning, the CPF-exposed rats were significantly heavier (by 13 g, on average; p<0.01) and longer (by 2 cm, on average; p<0.01) than non-exposed controls. No significant weight or length differences were observed at birth or at D60.

### Evaluation of intestinal permeability with FITC-dextran

**Figure 2:**
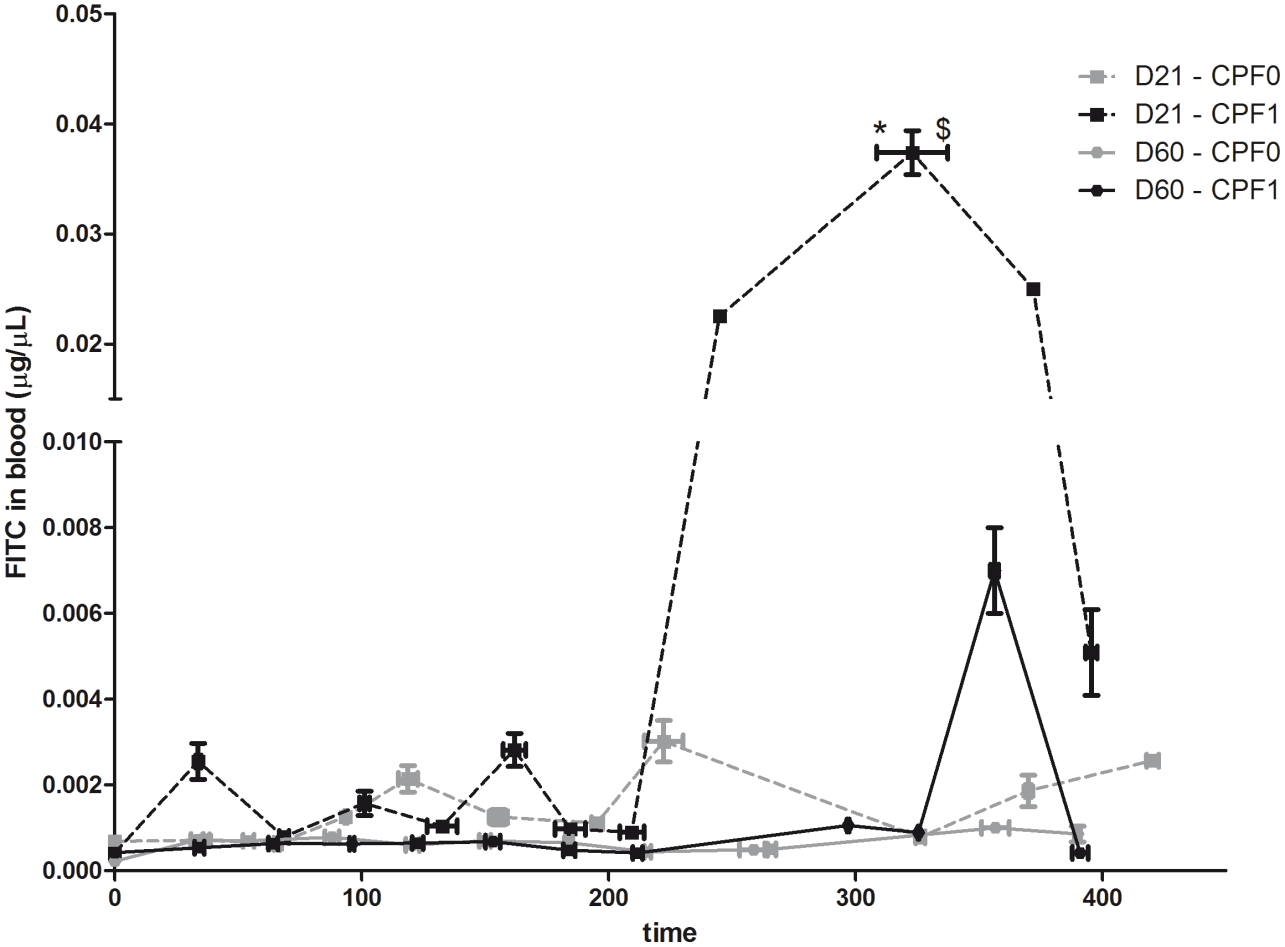
The time course of the appearance of FITC-dextran in blood (µg/µL) in control rats (CPF0, grey line) or CPF-exposed pups (CPF1, 1 mg/kg/day, black line) at weaning (D21) and as young adults (D60). Values are quoted as the mean ± SEM. D21: CPF0 n = 12, CPF1 n = 17; D60: CPF0 n = 14, CPF1 n = 13. * p<0.05.

The FITC-dextran-associated fluorescence peak was clearly observed in blood from CPF-exposed animals but not in blood from control animals (Figure 2). ThisCPF1 *vs.* CPF0difference was statistically significant at D21 (p<0.05). Moreover, FITC-dextran entered the blood quickly in CPF1 rats tested at D21. Comparison of the corresponding time points at D21 and D60 in the CPF-exposed groups revealed that FITC-dextran was first detected after323.0±14.4 min in D21 animals and after 356.3±1.7 min in D60 animals. Furthermore, FITC-dextran was detected in a significantly higher proportion at D21 than at D60 (p<0.05).

### Gene expression of TJ proteins

**Figure 3:**
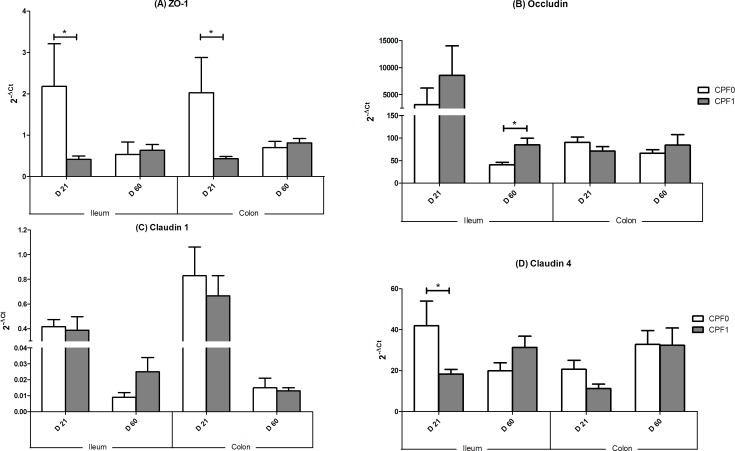
Gene expression (mRNA) of markers of intestinal maturation in the ileum and colon of control rats (CPF0, open bars) or CPF-exposed pups ((CPF1, 1 mg/kg/day, grey bars) at weaning (D21) and as young adults (D60), expressed as 2^−DCt^. Values are quoted as the mean ± SEM. D21: CPF0 n = 28, CPF1 n = 24; D60: CPF0 n = 21, CPF1 n = 13. * p<0.05. (A) ZO-1; (B) occludin; (C) claudin1; (D) claudin4.

We observed differences in gene expression between exposed and non-exposed pups (Figure 3).

At weaning, we observed a decrease in ZO-1 mRNA expression in ileum and colon (p<0.05 for both) in CPF1 animals compared to CPF0. Claudin 4 mRNA expression was lower in the ileum (p<0.05) in CPF1 animals than in CPF0 animals. Both claudin 1 and occludin did not display any variations in mRNA expression at D21.

At D60, no variation of mRNA expression was observed for ZO-1, claudin 1 and claudin 4. mRNA expression of occludin rose in ileum (CPF1 *vs.* CPF0, p<0.05).

### Immunohistochemical assessment of ZO-1 and claudin 4

**Figure 4:**
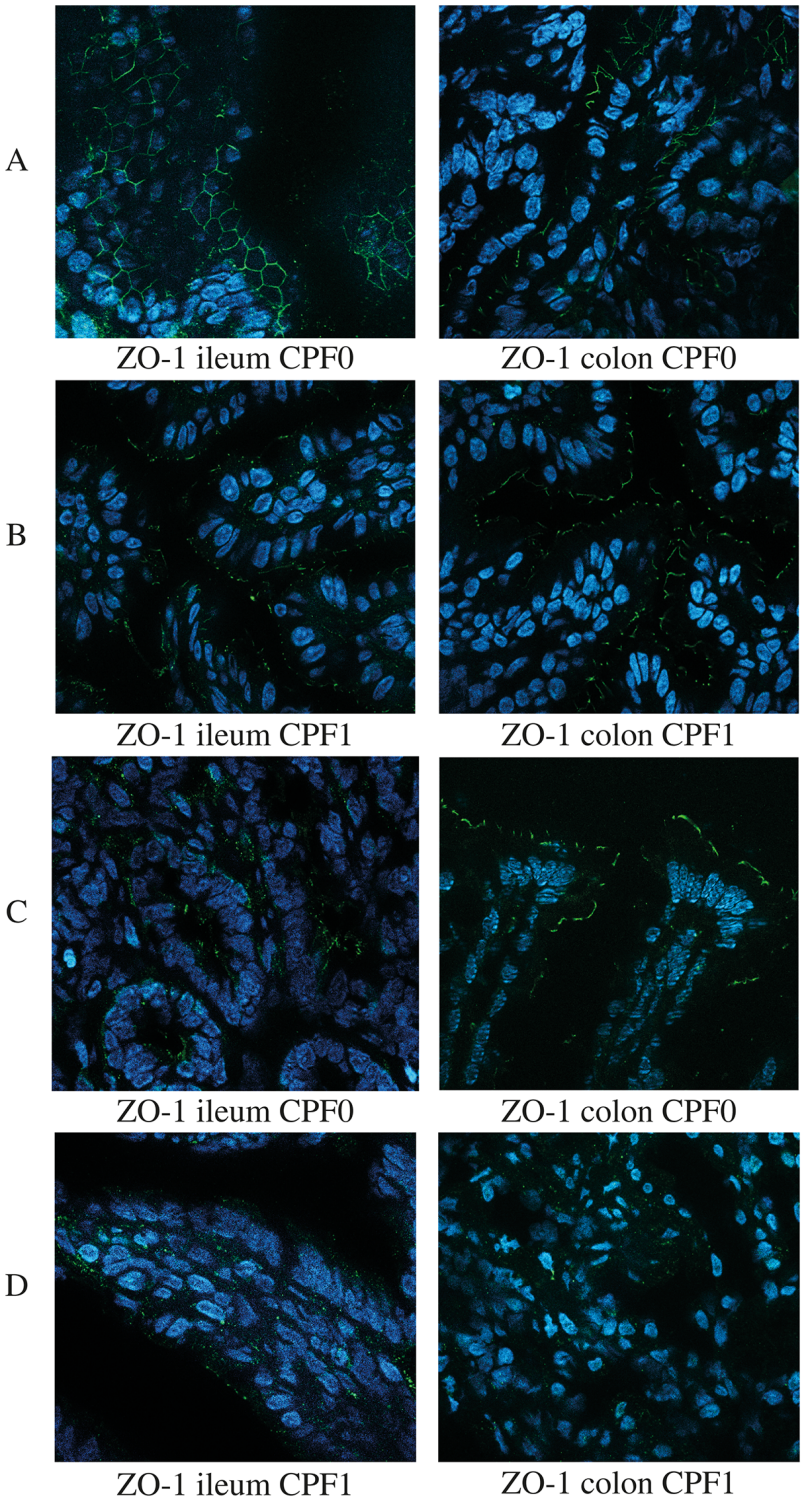
Immunofluorescent staining of ZO1 in the ileum and colon of CPF-exposed (1 mg/kg/day, CPF1) rats and control (CPF0) rats at weaning (D21) and as young adults (D60). Magnification: x 630. D21: CPF0 n = 5, CPF1 n = 5; D60: CPF0 n = 5, CPF1 n = 5. ZO-1 detection at D21 and D60 in CPF0rats (panels A and C, respectively) and in CPF1rats (panels, B and D respectively).

To investigate the distribution and localization of ZO-1 and claudin-4 in the gastrointestinal tract, we used rabbit polyclonal primary antibodies directed against epitopes in the proteins' C-terminal domains. No labeling was observed on control slides for which the primary antibody had been omitted. We found that ZO-1 and claudin-4 were expressed in the two intestinal segments studied (ileum and colon). In D21 animals, ZO-1 was found to be expressed in the ileum and colon (Figure 4-A). We observed that ZO-1 was located at the apical cell membrane and the lateral cell membrane in the tissues of CPF0 animals. The labeling appeared as a marked apical belt and well-defined points especially in the ileum and less marked in the colon (Figure 4-A). In CPF1 animals, ZO-1 staining appeared as a discontinuous belt and faint points in the ileum. The CPF0 and CPF1animals did not differ in terms of the distribution and intensity of labeling of ZO-1 in the colon (Figure 4-B).

Claudin-4 staining displayed a different distribution pattern, which was located more diffusely within the cells in the ileum and the colon. More intense TJ staining was observed in the ileum of CPF0 rats. In the ileum, claudin-4 was predominantly located in the microvillus surface of epithelial cells. At D21, the CPF0 and CPF1 animals did not differ clearly in terms of the distribution of claudin-4 in epithelial cells (data not shown).

In D60 rats, the CPF0 and CPF1 animals did not differ in terms of the distribution and intensity of labeling of ZO-1. In CPF0 rats, ZO-1 staining was barely visible and weak in the ileum. However, ZO-1 was clearly visible on top of villosities on the apical surface of colonic epithelial cells in CPF0 animals (Figure 4-C). In CPF1 animals, ZO-1 staining was weakly visible in the ileum and almost undetectable in the colon (Figure 4-D). As had been seen inCPF0 rats at D21, claudin-4 was essentially located in the cytoplasm of cells in this group at D60. In the ileum, the surface epithelium was strongly stained both in CPF0 and CPF1 rats. In the colon, claudin-4 staining was weak in CPF0 rats and barely visible in CPF1 rats (data not shown).

### Bacterial translocation

**Figure 5:**
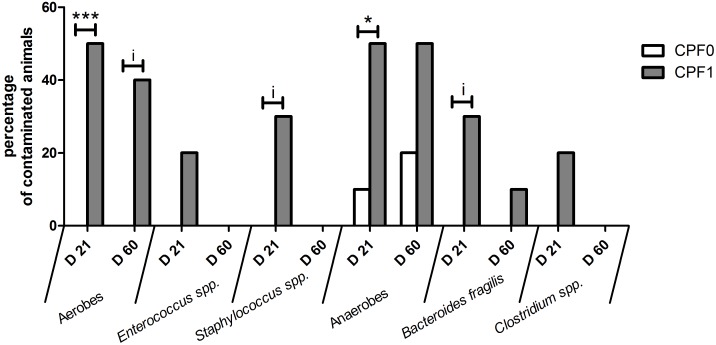
Percentage of rats contaminated by bacterial translocation (mean ± SEM) to the spleen at weaning (D21) and as young adults (D60). Control groups (CPF0, open bars) and CPF-exposed groups (CPF1: 1 mg/kg/day, grey bars). i, p<0.10; * p<0.05; ** p<0.01; *** p<0.001. D21: CPF0 n = 10, CPF1 n = 10; D60: CPF0 n = 10, CPF1 n = 10.

Aerobes and anaerobes were better able to translocate to the spleen (Figure 5) in CPF1 rats than in CPF0 rats (with contamination rates of up to 50% and up to 20%, respectively).

At weaning (D21), we observed greater bacterial translocation of total aerobes and total anaerobes in CPF1 pups than in CPF0 pups (50%, *vs.* 0%; p<0.001; 50% *vs*.10%; p<0.05, respectively). Concerning aerobes, *Enterococcus spp.* was found in 20% of the CPF1 pups (*vs.* 0% of the CPF0 pups) and *Staphylococcus spp.* was found in 30% of the CPF1 pups (*vs.* 0% of the CPF0 pups; p<0.10). For anaerobes, *Bacteroides fragilis* was found in 30% of the CPF1 pups (*vs.* 0% oftheCPF0 pups; p<0.10) and *Clostridium spp.* was found in 20% of the CPF1 pups(*vs.* 0% of the CPF0 pups).

At D60 (adulthood), the extent of translocation was lower than atD21 (weaning). Total aerobes were found in 40% of CPF1 rats (*vs.* 0% of the CPF0 pups, indicative, p<0.1) but neither *Enterococcus spp.* nor *Staphyloccus spp.* had translocated. Hence, the observed translocation must have been due to other species. We observed translocation of total anaerobes in 50% of the CPF1 animals (*vs.* 20% of CPF0 rats) and translocation of *Bacteroides fragilis* in 10%of the exposed-animals (*vs.* 0% of controls). Again, other species of bacteria must have contributed to this translocation.

## Discussion

After birth, maturation of the digestive barrier is particularly important because it enables (i) the gradual maturation of immune cells and (ii) prevents the occurrence of intolerance and adverse reactions to food allergens. Lactation is also crucial for the functional development of the digestive system. The progressive closure of the gut epithelium at weaning reflects the ability of the epithelial barrier's development as a very selective interface between the intestinal lumen and the internal environment [16].

In our permeability experiments, the progressive detection of 4 kDa FITC-dextran in the blood compartment is consistent with leakage from the lumen and suggests the presence of a change in paracellular permeability rather than in transcellular transport. The fact that elevated permeability was only observed in CPF-exposed animals indicates that chronic CPF exposure has a strong impact on the epithelial barrier - especially at weaning (D21), when the digestive system is still immature.

Changes in the expression of genes coding for TJ proteins were also observed at this time point. ZO-1 did not show any significant variation while a tendency in a reduction of mRNA expression was observed in immature CPF-treated animals (D21), both in ileum and colon. Such an effect of CPF was not present in mature animals. The impact of CPF was more striking in D21 rats by lowering mRNA expression of claudin 4. Low claudin expression may disrupt the protein complexes involved in the constitution of TJs.

These observations can be compared with the results of a previous study in which we observed that intestinal morphological structures are also affected by CPF-exposure [17]. We also found that CPF-exposure was associated with differences in protein expression in segments of the intestine. The immunohistochemical identification of claudin 4 seems to confirm Chiba et al.'s finding that claudin 4 is more strongly expressed in the small intestine than in the colon [18]. Accordingly, one can hypothesize that CPF has a putative downregulating effect on claudin 4.

Many studies have shown that disruption of TJs leads to a rise in epithelial permeability. For example, methotrexate (a drug used to treat cancer and autoimmune diseases) induces ZO-1 dephosphorylation, which in turn is associated with a change in the protein's localization in epithelial cells in the small intestine; this change may contribute to leakage of the intestinal barrier [19].

Here, we used immunofluorescent staining to study protein localizations in epithelium because changes in permeability may modify protein addressing or induce protein internalization. Both ZO-1 and claudin 4 are reportedly involved in intestinal barrier dysfunction [20], [21].

We detected ZO-1 and claudin 4 in the rat ileum and colon at both D21 and D60. Exposure to CPF was associated with changes in the expression and/or localization of ZO-1 both in the ileum and colon. Surprisingly, CPF did not seem to impact on the intestinal distribution of claudin 4 - at least in immature animals. In D60 animals, ZO-1 and claudin 4 staining was much weaker in CPF1 rats than in CPF0 rats; this might be due to downregulated expression and/or altered localization.

These observations could be related to Gearhart's study that showed that chlorpyrifos inhibit kinesin-dependent microtubule motility and Grigoryan's study that showed a tubulin polymerization disruption by chlorpyrifos oxon [22], [23]. As a consequence, proteins synthetized and involved in tight junction formation might be restrained to the cytoplasmic space rather than moving along microtubules to tight junction space.

The cholinergic signaling is also involved in the regulation of the barrier function. As described by Cameron and Perdue [24], HRP uptake as a marker of transepithelial transport is stimulated by the M3 muscarinic agonist bethanechol. In a similar way, the paracellular transport is also regulated by a cholinergic signaling. For example, carbachol, a cholinergic agonist, ameliorates LPS-induced intestinal epithelial tight junction damage by down-regulating NF-κB and myosin light-chain kinase pathways [25]. Chlorpyrifos oxon is known to inhibit acetylcholinesterase activity [26] which increases the amount of acetylcholine in the synaptic space. Consequently, we could suspect an overstimulation of acetylcholine receptors leading to a rise in nerve transmission. But CPF oxon has been described to block muscarinic receptors [27], reducing the potentially protective effect of acetylcholine on gut barrier dysfunction.

The results of our previous study of rat pups revealed that chronic CPF-exposure was also associated with a microbial dysbiosis at weaning (characterized by the proliferation of potentially harmful bacteria) [14]. The same phenomenon was observed for mature animals at 60 days of age, and was associated with greater bacterial translocation to the liver [17]. Bacterial toxins may be involved in the disruption of the cytoskeleton and thus leakage of macromolecules through the paracellular route [28]. Moreover, CPF-induced dysbiosis was characterized by low counts of lactobacilli [14], which (under normal conditions) are involved in the relocation of occludin and ZO-1 to TJ complexes[29]. In view of the fact that many foodstuffs are contaminated with pesticide residues, consumption of probiotics may help prevent dysbiosis and protect TJs [30]. Dysbiosis and bacterial translocation may also stimulate the immune system and trigger the release of cytokines; this may also be a strong signal for the disruption of cell-cell contacts. These cytokines can directly affect cell-cell contacts and paracellular permeability in intestinal epithelial cells by modulating claudin expression and distribution [31].

Bacterial translocation is known to contribute to the development of inflammation and various functional gastrointestinal disorders (such as irritable bowel syndrome and celiac disease) and chronic inflammatory diseases (such as Crohn's disease) [2], [32]. It is also recognized that microbial dysbiosis exerts a strong influence on the immune system and intestinal permeability [33], [34].

On the basis of our present results, it is impossible to say whether CPF's effect is direct or indirect. As already mentioned above, CPF may modify the function of the enteric nervous system and its associated cholinergic signaling, which in turn may modulate epithelial barrier permeability in general and ZO-1 in particular [35]. In the present study, animals were constantly exposed to CPF. It would have been interesting to see whether CPF-associated changes were transient or permanent. Indeed, Yeh et al. reported that chitosan-induced changes in TJs were reversible when exposure to the toxic substance ceased [36]. Overall, CPF-exposure can be considered to be a stressor; other studies have found that stress disrupts intestinal homeostasis and increases the permeability of the rat ileum [31].

## Conclusions

Under physiological conditions, the digestive tract's epithelium serves as a strong, selective barrier against potentially harmful bacteria and food allergens. Children with asymptomatic food allergies have abnormally high intestinal permeability [37].

Chronic CPF-exposure during critical pre- and postnatal periods of organ development and maturation alters epithelial barrier function, which in turn is associated with elevated permeability and bacterial translocation. Furthermore, the barrier dysfunction is associated with changes in TJ protein expression.

In summary, pesticide residues in food may impact the digestive tract function and its ability to adapt to environmental changes. In rats, this effect appears to be even greater at the time of weaning (i.e. when food diversification occurs). Our data highlight the impact of food contaminants on the digestive system - especially in developing individuals.
